# Unexpected Hazards, Unanticipated Risks

**DOI:** 10.3201/eid2908.AC2908

**Published:** 2023-08

**Authors:** Byron Breedlove

**Affiliations:** Centers for Disease Control and Prevention, Atlanta, Georgia, USA

**Keywords:** art science connection, emerging infectious diseases, art and medicine, about the cover, public health, climate change, One Health, medical tourism, emerging and reemerging diseases, parasites, bacteria, vector-borne diseases, Alexis Rockman, Ark, Unexpected Hazards, Unanticipated Risks

**Figure:**
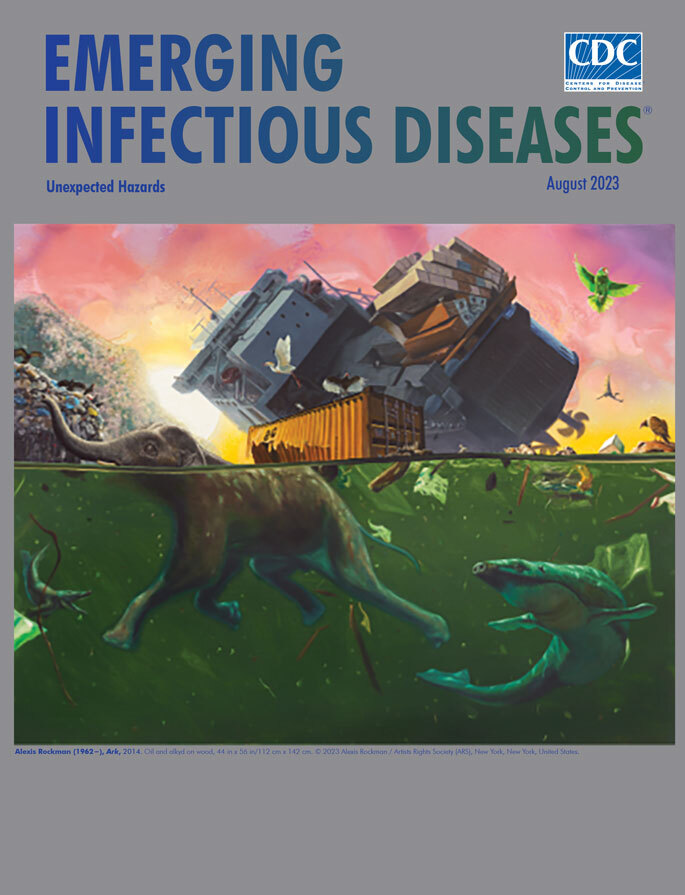
**Alexis Rockman (1962−), *Ark,* 2014.** Oil and alkyd on wood, 44 in x 56 in/112 cm x 142 cm. © 2023 Alexis Rockman/Artists Rights Society (ARS), New York, New York, USA.

Contemporary American artist Alexis Rockman was born and raised in New York City and studied animation at the Rhode Island School of Design and fine arts at the School of Visual Arts in Manhattan. His meticulously detailed paintings, which often depict ecosystems transformed by climate change, invasive species, and human activity, are found in public and private collections around the world. The Princeton University Art Museum states: “The artist’s vivid series of large canvases and intimate watercolors points to how an increasingly interconnected world has generated profound ecological change. Rockman is among the most accomplished contemporary eco-artists, having for several decades examined issues at the nexus of natural history, climate change, and biodiversity.” 

Those same issues mirror in large degree the central thesis of One Health, which stresses interconnections among the health of humans, animals, plants, and their shared environment. One Health issues include emerging and reemerging zoonotic diseases, neglected tropical diseases, vectorborne diseases, antimicrobial resistance, and environmental contamination. According to a 2022 analysis published in *Nature Climate Change* by Camilo Mora and other environmental scientists, humans are now more likely to come in contact with a broader range of infectious agents than ever before. Sometimes infectious diseases are spread by new or unexpected routes, including environmental sources, medical tourism, contaminated food and water from sources thought to be safe, or expansion of disease vectors into new areas. 

Rockwell’s painting *Ark*, featured on this month’s cover, shows the aftermath following the capsize of a cargo vessel laden with animals and provides a visual touchstone for contemplating the jarring impact of unexpected calamity. This disaster may have been triggered by instability from the ship’s being overloaded, collision with underwater wreckage, or rough waters spawned by a cyclone. The unnatural reddish glare of the sky contrasts with the lurid green polluted water teeming with mutated creatures, and together with a glimpse of land crusted with plastic and other debris reveal a ravished environment. Many of the animals being ferried to safer havens have been thrown into the water. Perhaps the elephant will make it to land, but others such as the moose and camels bobbing on the surface will likely drown or fall prey to the aquatic predators. Still clinging to the ship are a rhinoceros, leopard, panda, polar bear, and various other animals, alive but trapped. 

*Ark *serves as a potent reminder of best laid plans gone awry and countermeasures thwarted. Its inherent metaphor is perhaps analogous to the realm of infectious diseases. Despite having improved diagnostics and treatments, effective and safe vaccines, and interventions to mitigate health threats, it is not smooth sailing for public health professionals challenged by known issues such as an underfunded infrastructure and by an unexpected rise in the spread and acceptance of misinformation as fact. Continued support for laboratory and epidemiology resources needed for ongoing surveillance of emerging and reemerging infectious disease threats of the future remain crucial to maintain strong, stable public health systems.

Note: EID has previously featured artwork by Alexis Rockman on its April 2006 and May 2009 covers. 
